# Dietary Acidifier Modulates the Gut Microbiota–Metabolic Axis to Improve Intestinal Health, Yolk Fatty Acid Profiles, and Egg Quality in Laying Hens

**DOI:** 10.3390/ijms27156859

**Published:** 2026-07-30

**Authors:** Xiaotong Li, Qiaoqiao Han, Yaru Wang, Yue Wang, Zuowei Li, Sheng Wang, Wei Fu, Huiying Li, Haihua Zhang, Donghui Shi, Shimeng Huang

**Affiliations:** 1 State Key Laboratory of Animal Nutrition and Feeding, College of Animal Science and Technology, China Agricultural University, Beijing 100193, China; lixiaotong1117@163.com (X.L.); hanqq12306@163.com (Q.H.); wyrsmile0930@163.com (Y.W.); wangy07224@163.com (Y.W.); 15684302290@163.com (Z.L.); taofu12138@163.com (S.W.); 17660458161@163.com (W.F.); huiyang-1@outlook.com (H.L.); 2 Laboratory of Feed Grain Safety and Healthy Poultry Farming, Beijing Jingwa Agricultural Science and Technology Innovation Center, Beijing 101206, China; 3 College of Animal Science and Veterinary Medicine, Jinzhou Medical University, Jinzhou 121001, China; kathysdh@163.com; 4 Hebei Key Laboratory of Specialty Animal Germplasm Resources Exploration and Innovation, College of Animal Science and Technology, Hebei Normal University of Science and Technology, Qinhuangdao 066004, China; zhh83@126.com

**Keywords:** acidifier, egg quality, laying hens, lipid metabolism, yolk fatty acid composition, gut microbiome

## Abstract

Acidifiers are functional feed additives that may improve egg quality by modulating intestinal microbiota and host metabolism. This study investigated the effects of dietary acidifier supplementation on laying performance, yolk fatty acid composition, eggshell ultrastructure, intestinal function, cecal microbiota, serum metabolome, and antioxidant status in laying hens. Sixty 52-week-old Jingfen No. 6 laying hens were randomly assigned to either a control group or an acidifier-supplemented group for 6 weeks. Acidifier supplementation increased laying rate and egg mass while reducing the feed-to-egg ratio. It also increased the concentrations of seven yolk fatty acids, including tetradecanoic acid, myristoleic acid, palmitic acid, palmitoleic acid, stearic acid, cis-vaccenic acid, and arachidic acid. In addition, acidifier supplementation resulted in a denser eggshell microstructure, improved cecal mucosal morphology, and enhanced the activities of lipase, chymotrypsin, amylase, and trypsin. Although cecal microbial alpha diversity remained unchanged, acidifier supplementation altered the overall microbial community structure and enriched several genera, including *Slackia_A*, *Megasphaera_A*, *Veillonella_A*, *Merdimonas*, and *Caccocola*. Serum metabolomic analysis identified 395 differential metabolites, primarily associated with lipid, amino acid, energy, and bile acid metabolism. Representative altered metabolites included taurodeoxycholic acid 3-glucuronide, LysoPE (0:0/20:0), 15-HETE, cholic acid, malic acid, and succinic acid. Acidifier supplementation also favorably affected serum HDL-C, LDL-C, and calcium concentrations and enhanced antioxidant capacity. Collectively, these findings indicate that dietary acidifier supplementation improves laying performance, yolk fatty acid deposition, eggshell structure, intestinal function, and antioxidant status, accompanied by alterations in cecal microbiota and serum metabolism. These coordinated changes may contribute to the observed improvements in egg quality.

## 1. Introduction

As an economical and nutrient-dense source of high-quality protein, eggs provide essential amino acids, fatty acids, minerals, vitamins, and bioactive compounds required for human health [1]. Egg quality is a critical determinant of consumer acceptance, market competitiveness, and profitability in the poultry industry, and it is closely tied to the substantial scale of the egg sector within the global animal protein economy; even a modest proportion of egg breakage or quality deterioration (approximately 2% of global production) can cause significant economic losses [2,3]. With the increasing global demand for high-quality and value-added eggs, innovative and sustainable nutritional strategies are urgently needed to improve egg quality and nutritional composition, particularly through the modulation of yolk nutrient deposition and eggshell integrity [4,5].

Egg yolk, which accounts for approximately 30% of total egg mass, is especially rich in phospholipids, antioxidants, and diverse fatty acids with important physiological functions [6,7,8,9]. Polyunsaturated fatty acids (PUFAs), particularly n-3 fatty acids such as α-linolenic acid, eicosapentaenoic acid, and docosahexaenoic acid, play important roles in anti-inflammatory regulation, cardiovascular protection, and neural development, whereas monounsaturated fatty acids (MUFAs) are associated with improved lipid profiles and reduced cardiovascular risk [10,11]. The fatty acid composition of egg yolk is highly responsive to dietary manipulation; thus, nutritional intervention can selectively enhance the deposition of functional fatty acids, yielding eggs with superior nutritional value [7,12]. As the market for functional foods continues to expand, optimizing dietary approaches to modulate yolk fatty acid profiles has emerged as a key priority in poultry nutrition [4,13].

Over the past decade, dietary acidifiers have attracted growing interest as promising substitutes for antibiotic growth promoters in poultry production [14]. Their proposed mechanisms of action are multifaceted. Organic acids such as formic, propionic, acetic, and lactic acids can lower gastrointestinal pH, thereby inhibiting acid-sensitive pathogens, including *Salmonella* and *Escherichia coli*, while favouring the proliferation of beneficial acid-tolerant microorganisms like *Lactobacillus* and *Clostridium butyricum* [15]. Acidifiers may also improve feed palatability and stimulate pancreatic enzyme secretion, thereby enhancing nutrient digestibility [16,17]. In addition, ammonium salts such as ammonium formate and ammonium propionate present in acidifier formulations can release free acids under acidic conditions, further potentiating their antimicrobial effects, while simultaneously serving as non-protein nitrogen sources that participate in host nitrogen metabolism [18,19,20]. Furthermore, certain organic acids act as metabolic substrates and signalling molecules that influence energy metabolism, lipid homeostasis, and antioxidant capacity [17,21,22]. More specifically, propionate suppresses fatty acid synthase activity, curbs de novo lipogenesis, and promotes fatty acid oxidation via activation of the AMPK signalling pathway [23,24]. Formic acid, in turn, participates in one-carbon metabolism through the formate dehydrogenase pathway, affecting nucleotide and amino acid biosynthesis [25].

Several studies have demonstrated that acidifier mixtures can improve laying performance, eggshell quality, and intestinal morphology in laying hens [17,26,27,28]. Recent studies have also shown that acidifiers can modulate gut microbiota, increasing the abundance of short-chain fatty acid-producing genera and indirectly influencing host immunity and metabolism [29,30]. However, most of these studies have focused on production performance and gross egg quality parameters, with limited attention to yolk fatty acid deposition and its underlying mechanisms. In particular, how acidifiers influence yolk fatty acid composition through the gut microbiota–host metabolic axis, and the potential synergistic or antagonistic effects among different organic acids, remain poorly understood [31].

We hypothesized that dietary acidifier supplementation is associated with improvements in egg quality and nutritional value, potentially linked to changes in yolk fatty acid profiles, eggshell structural integrity, and the gut microbiota–host metabolic axis. Therefore, the objective of this study was to systematically evaluate the associations between acidifier supplementation and production performance, egg quality, yolk fatty acid composition, eggshell ultrastructure, intestinal health, gut microbiota composition, and related metabolic responses in laying hens. This study aimed to explore the potential interplay among dietary acidifiers, the gut microbiota, and host metabolism, with the goal of generating hypotheses and providing a foundation for future mechanistic investigations, as well as informing the development of sustainable nutritional strategies for improving egg quality and producing value-added eggs.

## 2. Results

### 2.1. Fatty Acid Composition of Egg Yolk

**Figure 1:**
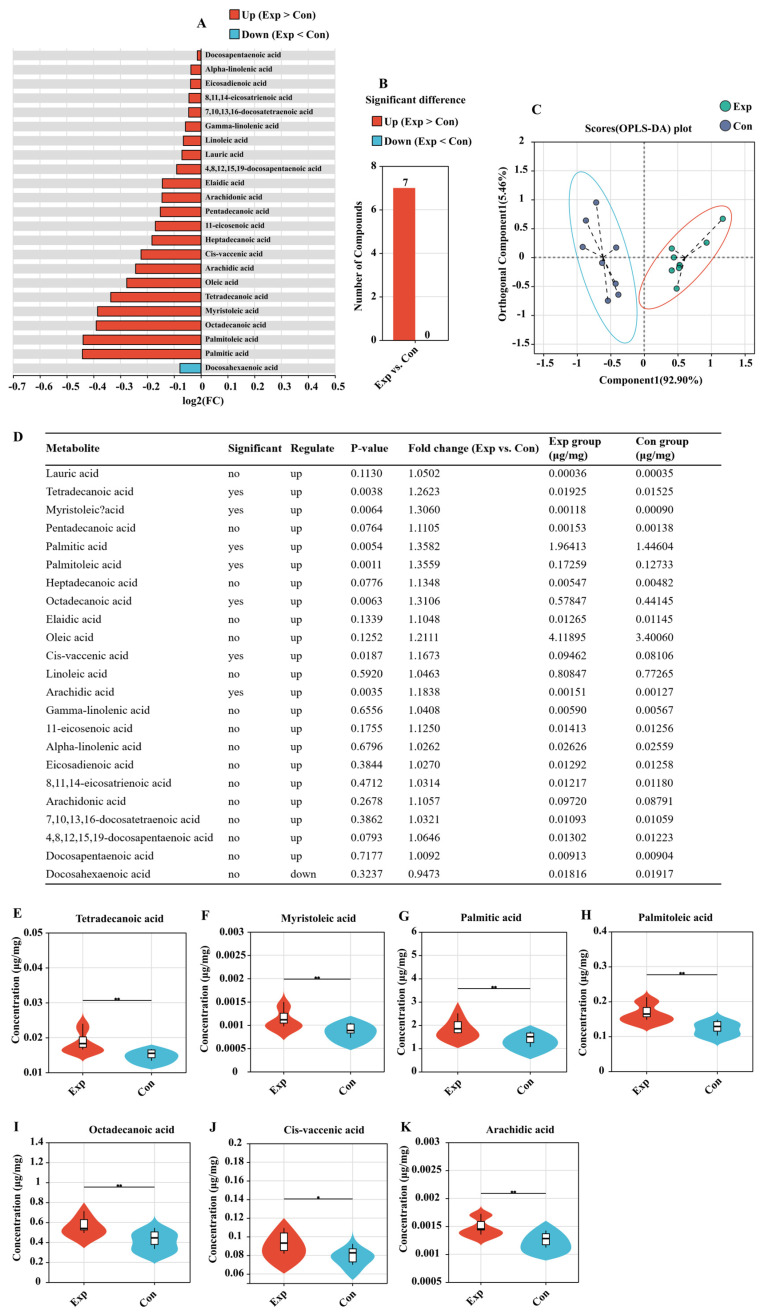
Acidifier affects the fatty acid composition of egg yolks in laying hens. Significant differences in fatty acid metabolites between the Con and Exp groups are indicated by (**A**) LEfSe, (**B**) Number of differential fatty acids, with red representing upregulated and blue indicating downregulated between the groups, (**C**) Scores (OPLS-DA) Plot, and (**D**) table of significance of fatty acid metabolites, respectively. Changes in the concentration of seven fatty acids in egg yolks: (**E**) Tetradecanoic acid; (**F**) Myristoleic acid; (**G**) Palmitic acid; (**H**) Palmitoleic acid; (**I**) Octadecanoic acid; (**J**) Cis-vaccenic acid; (**K**) Arachidic acid. * *p* < 0.05, ** *p* < 0.01.

The medium- and long-chain fatty acid profiles of egg yolk were determined by gas chromatography–mass spectrometry (GC–MS) after derivatization to fatty acid methyl esters. A total of 23 fatty acids were included in the linear discriminant analysis effect size (LEfSe) analysis. Among them, 22 fatty acids were enriched in the Exp group, whereas docosahexaenoic acid was decreased compared with the Con group (*p* < 0.05; Figure 1A). Orthogonal partial least squares discriminant analysis (OPLS-DA) showed clear separation between the Con and Exp groups, indicating distinct yolk fatty acid profiles following acidifier supplementation (Figure 1C). Compared with the Con group, seven fatty acids were significantly increased in the Exp group, including tetradecanoic acid, myristoleic acid, palmitic acid, palmitoleic acid, octadecanoic acid, cis-vaccenic acid, and arachidic acid (*p* < 0.05; Figure 1B,D). Consistently, quantitative analysis further confirmed that dietary acidifier supplementation significantly increased the concentrations of these seven fatty acids in egg yolk (*p* < 0.05; Figure 1E–K).

### 2.2. Ultrastructure of the Eggshell

**Figure 2:**
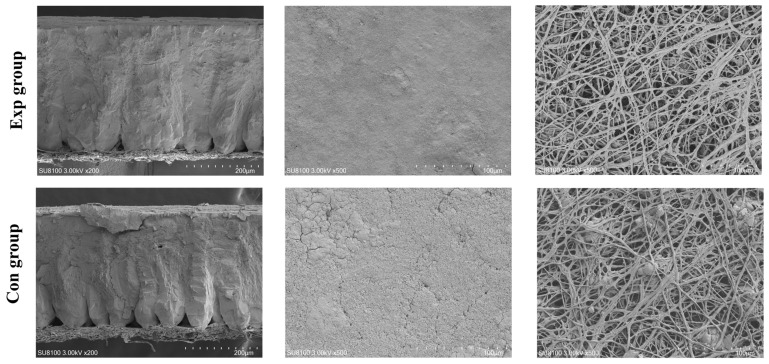
Acidifier improves eggshell structure in laying hens. 3.00 kv × 200 μm and 3.00 kv × 500 μm per image.

Eggshell ultrastructure was examined by scanning electron microscopy (SEM) to characterize the microscopic architecture and spatial organization of the eggshell matrix. As shown in Figure 2, representative cross-sectional SEM images obtained at 3000× magnification revealed marked structural differences between the Con and Exp groups. Eggshells from the Exp group exhibited a denser and more uniform microstructure, characterized by a compact and well-organized interconnected network. In contrast, eggshells from the Con group displayed a relatively porous morphology, with a looser and less compact network structure. These observations suggest that dietary acidifier supplementation improved eggshell structural integrity.

### 2.3. Cecal Histomorphology and Digestive Enzyme Activity

**Figure 3:**
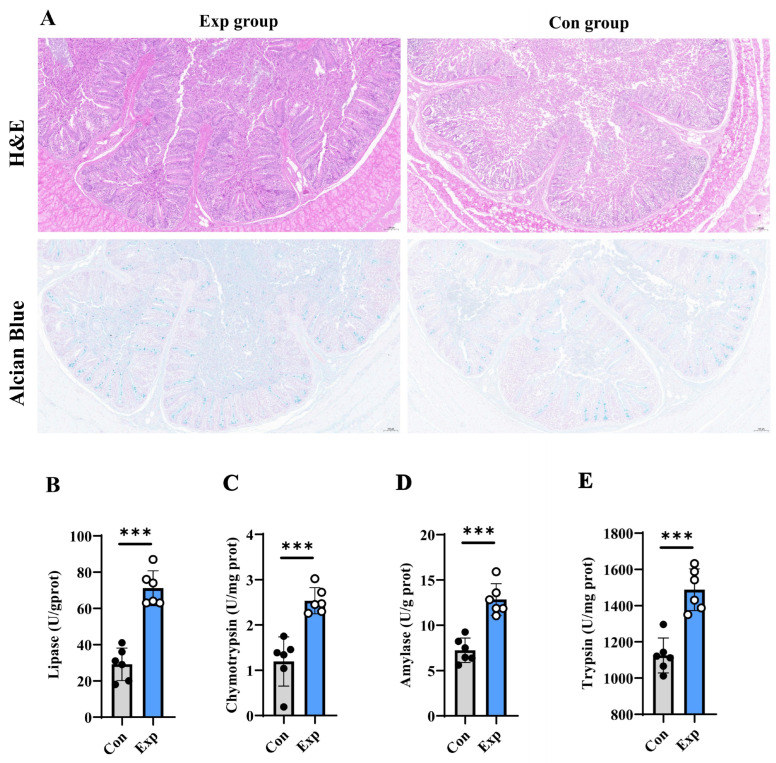
Acidifier improves intestinal function in laying hens. The changes in (**A**) representative H&E and Alcian Blue staining images of hen cecum at 40 magnification (Scale bar = 100 µm) and (**B**–**E**) digestive enzyme activities. *** *p* < 0.001.

Histological analysis revealed apparent morphological differences in the cecum between the Con and Exp groups (Figure 3A). Compared with the Con group, the Exp group showed more regularly arranged cecal mucosal structures, with compact mucosal folds and a thicker lamina propria. The mucosal surface also appeared more consolidated, suggesting improved cecal tissue organization following acidifier supplementation. Cecal digestive enzyme activities were further assessed. As shown in Figure 3B–E, dietary acidifier supplementation significantly increased the activities of lipase, chymotrypsin, amylase, and trypsin compared with the Con group (*p* < 0.001). These results indicate that acidifier supplementation enhanced digestive enzyme activity in the cecum.

### 2.4. Cecal Microbiota Analysis in Laying Hens

**Figure 4:**
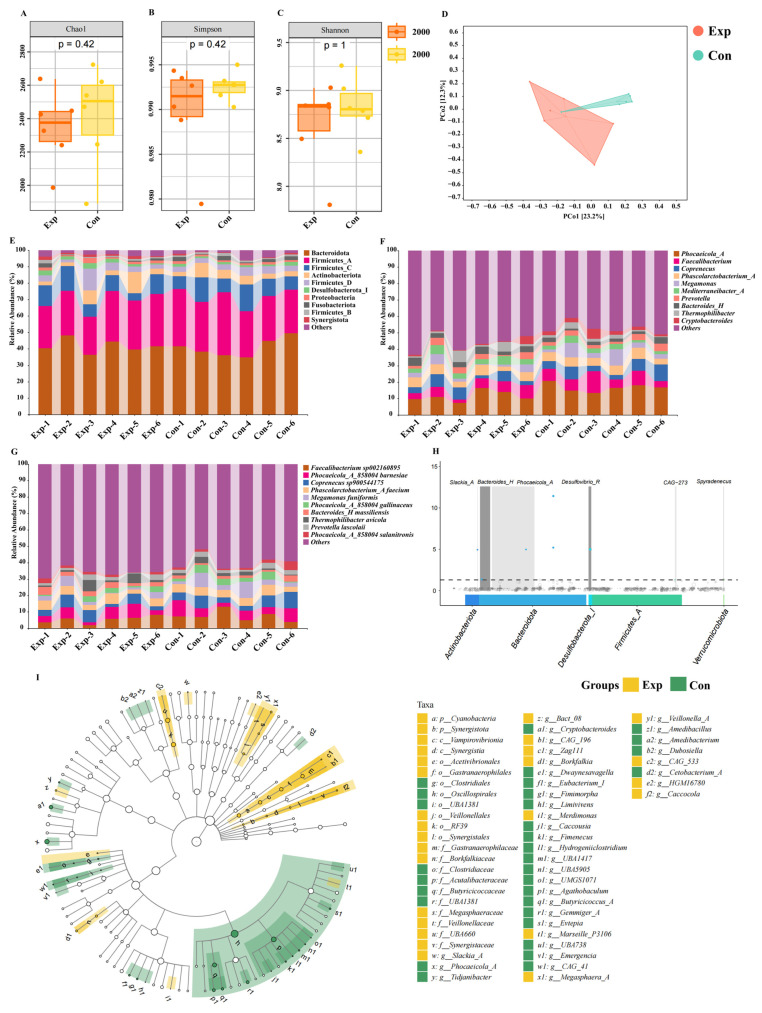
Effect of acidifier on microbiological analysis of the cecum of laying hens. Alpha diversity analysis was compared according to the microbial diversity index: (**A**) Chao 1, (**B**) Simpson, and (**C**) Shannon. The beta diversity: (**D**) principal coordinates analysis (PCoA). The microbial community structure: (**E**) the phylum level, (**F**) the family level, (**G**) the genus level. (**H**) Correlation analysis of bacterial flora. (**I**) generated from LEfSe analysis representing multiple taxa differentially enriched in the cecal samples from the Exp group (yellow) and Con group (green) hens (*p* < 0.05, LDA > 2).

The effects of dietary acidifier supplementation on cecal microbial diversity were evaluated using 16S rRNA gene sequencing. Alpha diversity, as assessed by the Chao1, Simpson, and Shannon indices, did not differ significantly between the Con and Exp groups (Figure 4A–C). In contrast, principal coordinate analysis (PCoA) based on Bray–Curtis dissimilarity showed clear separation between the two groups, indicating that acidifier supplementation altered the overall structure of the cecal microbiota (Figure 4D). Microbial composition was subsequently analyzed at different taxonomic levels. At the phylum level, Bacteroidota and Firmicutes were the dominant phyla in both groups and together accounted for more than 60% of the total bacterial community (Figure 4E). At the family level, *Phocaeicola_A* and *Faecalibacterium* were the predominant taxa in both groups, although their relative abundances were lower in the Exp group than in the Con group (Figure 4F). At the genus level, *Faecalibacterium*, *Phocaeicola_A*, *Coprenecus*, *Phascolarctobacterium_A faecium*, and *Megamonas_A* were the most abundant bacterial species in both groups (Figure 4G). In addition, Actinobacteriota, Bacteroidota, Desulfobacterota_I, Firmicutes_A, and Verrucomicrobiota were identified as the dominant phyla contributing to the cecal microbial community structure in the Con and Exp groups (Figure 4H). LEfSe analysis was performed to identify discriminative bacterial taxa between the two groups (*p* < 0.05, LDA score > 2). As shown in Figure 4I, several genera were significantly enriched in the Exp group, including *Slackia_A*, *Bact_08*, *CAG_196*, *Borkfalkia*, *Zaglll*, *Merdimonas*, *Marseille_P3106*, *Megasphaera_A*, *Veillonella_A*, *CAG_533*, *Caccocola*, and *HGM16780*. These findings indicate that dietary acidifier supplementation selectively reshaped specific cecal microbial taxa without markedly affecting alpha diversity.

### 2.5. Untargeted Metabolomics Analysis

**Figure 5:**
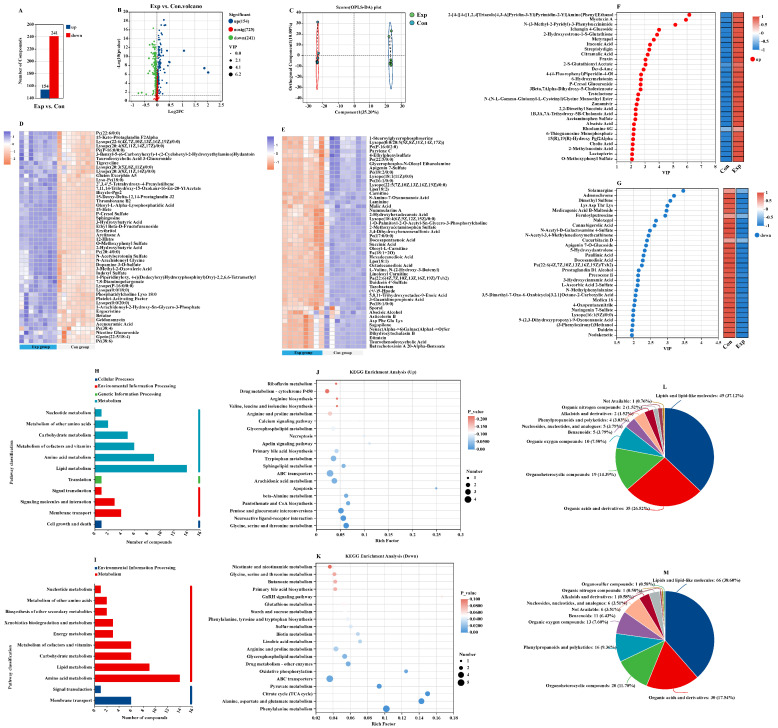
Serum Untargeted Metabolomics of laying hens. (**A**) Number of differential metabolites, with blue representing upregulated and red indicating downregulated metabolites between the groups; (**B**) Volcano Plot; (**C**) Scores (OPLS-DA) Plot; (**D**) Heatmap showing relative abundance of upregulated differential metabolites; (**E**) Heatmap showing relative abundance of downregulated differential metabolites; (**F**) VIP ranking plot of serum upregulated differential metabolites; (**G**) VIP ranking plot of serum downregulated differential metabolites; (**H**) Pathway classification bar chart for upregulated metabolites; (**J**) Bubble plot of KEGG enrichment analysis for upregulated metabolites; (**L**) Pie chart illustrating the relative abundance composition of serum upregulated metabolites between Con and Exp groups; (**I**) Pathway classification bar chart for downregulated metabolites; (**K**) Bubble plot of KEGG enrichment analysis for downregulated metabolites; (**M**) Pie chart illustrating the relative abundance composition of serum downregulated metabolites between Con and Exp groups.

To further characterize systemic metabolic alterations induced by dietary acidifier supplementation, untargeted LC–MS/MS-based metabolomics analysis was performed using serum samples from laying hens (Figure 5). In total, 395 significantly differential metabolites were identified in the Exp group compared with the Con group, including 154 upregulated and 241 downregulated metabolites (Figure 5A,B). OPLS-DA revealed clear clustering and separation between the two groups, indicating distinct serum metabolic profiles following acidifier supplementation (Figure 5C). Heatmaps visualized the abundance variation in the top 50 upregulated and top 50 downregulated differential metabolites across the two experimental cohorts (Figure 5D,E). Relative to the Con group, multiple metabolites including taurodeoxycholic acid 3-glucuronide, LysoPE (0:0/20:0), bicyclo-PGE_2_ and 15-HETE were significantly accumulated in the Exp group. On the contrary, the levels of 4-ethylphenyl sulfate, (±)9-HPODE, 5,8,11-trihydroxyoctadecenoic acid, malic acid and succinic acid were markedly reduced. Variable importance in projection (VIP) screening highlighted the core elevated metabolites in Exp serum, namely 2-S-glutathionyl acetate, p-cresol glucuronide, acetaminophen sulfate, N-(N-L-γ-glutamyl-L-cysteinyl)glycine monoethyl ester, 15(R),19(R)-hydroxy PGF_2_α and cholic acid (Figure 5F). Meanwhile, adrenochrome, apigenin 7-O-glucoside, dimethyl sulfone, naloxegol and 5-hydroxycinnamic acid were the top metabolites with reduced abundance in the Exp group (Figure 5G). Global pathway classification indicated that all differential metabolites participated in diverse biological functional categories, including cellular processes, environmental information processing, genetic information processing and core metabolic pathways, with prominent enrichment in lipid and amino acid metabolic cascades (Figure 5H,I). Subsequent Kyoto Encyclopedia of Genes and Genomes (KEGG) enrichment bubble plot analysis revealed that upregulated metabolites were primarily enriched in tryptophan metabolism, sphingolipid metabolism, arachidonic acid metabolism, pentose and glucuronate interconversions, neuroactive ligand–receptor interaction, as well as glycine, serine and threonine metabolism pathways (Figure 5J). By contrast, pathways suppressed upon acidifier supplementation contained drug metabolism—other enzymes, pyruvate metabolism, the tricarboxylic acid (TCA/citrate) cycle, oxidative phosphorylation, alanine/aspartate/glutamate metabolism and phenylalanine metabolism (Figure 5K). Chemical structural classification pie charts illustrated that lipids and lipid-like molecules constituted the dominant category for both upregulated and downregulated metabolites, followed by organic acids and derivatives, as well as organoheterocyclic compounds (Figure 5L,M). Taken together, these metabolomic data confirm that dietary acidifier supplementation profoundly remodels serum metabolic landscapes of laying hens, predominantly modulating lipid metabolism, amino acid metabolism and energy homeostasis-related pathways.

### 2.6. Laying Performance and Serum Indices

**Figure 6:**
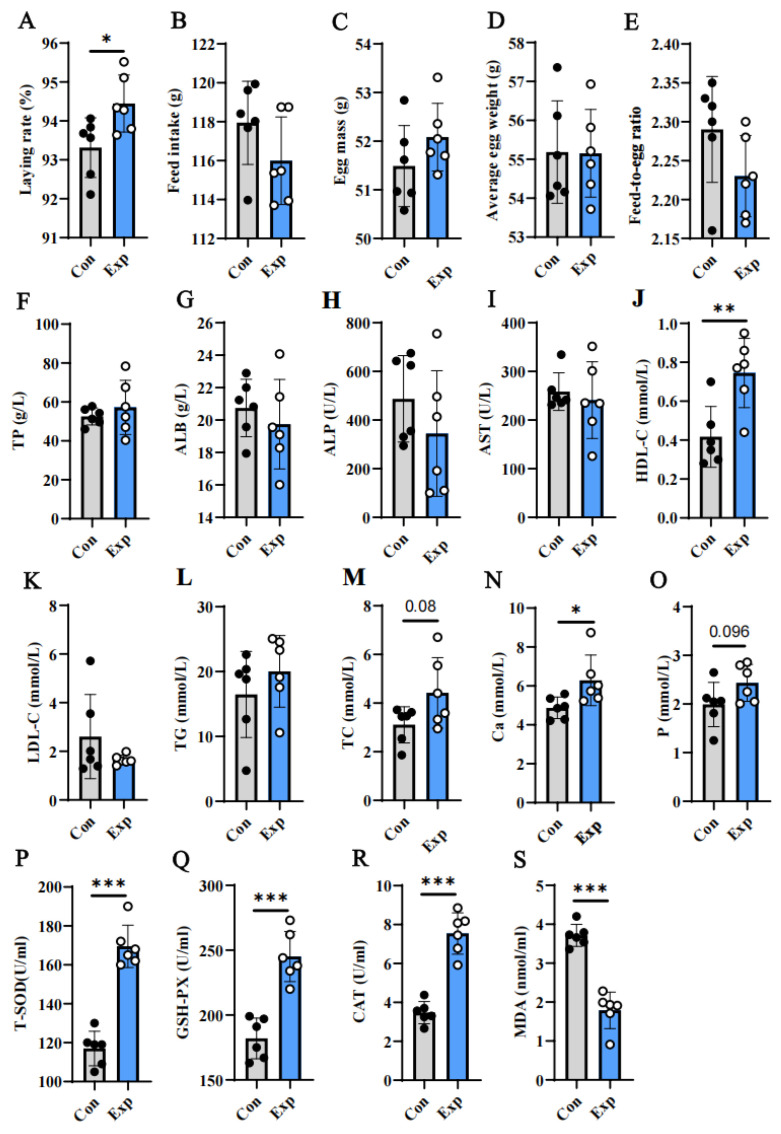
Effect of adding acidifier to the diet of laying hens on production performance. Laying performance: (**A**) Laying rate, (**B**) Feed intake, (**C**) Egg mass, (**D**) Average egg weight, (**E**) Feed-to-egg radio. Serum biochemical indicators: (**F**) TP, (**G**) ALB, (**H**) ALP, (**I**) AST, (**J**) HDL-C, (**K**) LDL-C, (**L**) TG, (**M**) TC, (**N**) Ca, (**O**) P. Serum antioxidant indices: (**P**) T-SOD, (**Q**) GSH-PX, (**R**) CAT, (**S**) MDA. * *p* < 0.05, ** *p* < 0.01, *** *p* < 0.001.

The effects of dietary acidifier supplementation on laying performance are shown in Figure 6. Compared with the Con group, the Exp group exhibited a significantly higher laying rate (*p* < 0.05; Figure 6A), whereas average egg weight was not significantly affected (*p* > 0.05; Figure 6D). Feed intake and the feed-to-egg ratio were reduced in the Exp group compared with the Con group (Figure 6B,E). In addition, egg mass was increased following acidifier supplementation (Figure 6C), indicating improved productive performance in laying hens. Serum biochemical indices were further analyzed to evaluate the effects of acidifier supplementation on metabolic and physiological status. Compared with the Con group, acidifier supplementation significantly increased serum high-density lipoprotein cholesterol (HDL-C) levels and decreased low-density lipoprotein cholesterol (LDL-C) levels (*p* < 0.05; Figure 6J,K). Serum total protein (TP) levels were higher in the Exp group, whereas albumin (ALB), alkaline phosphatase (ALP), and aspartate aminotransferase (AST) levels were lower than those in the Con group; however, these differences were not statistically significant (*p* > 0.05; Figure 6F–I). Serum triglyceride (TG) and total cholesterol (TC) levels were also increased in the Exp group (Figure 6L,M). In addition, dietary acidifier supplementation significantly increased serum calcium levels (*p* < 0.05; Figure 6N) and tended to increase serum phosphorus levels, although the difference was not statistically significant (*p* > 0.05; Figure 6O). Regarding antioxidant status, the Exp group showed significantly higher activities of total superoxide dismutase (T-SOD), glutathione peroxidase (GSH-PX), and catalase (CAT), accompanied by a marked decrease in malondialdehyde (MDA) levels (*p* < 0.001; Figure 6P–S). These findings indicate that dietary acidifier supplementation improved laying performance, lipid-related serum indices, mineral status, and antioxidant capacity in laying hens.

## 3. Discussion

Eggs are a high-quality dietary protein source, and both their internal nutritional value and external structural integrity are key determinants of consumer acceptance and market value [32,33,34]. While eggshell quality reflects physical protection, yolk composition—particularly its lipid profile—plays a central role in nutritional functionality [35]. Nevertheless, substantial economic losses persist across the egg production chain due to breakage and quality deterioration during collection, transportation, and storage, processes closely associated with eggshell strength and the physiological status of laying hens [36,37]. Previous studies have demonstrated that dietary acidifier supplementation can improve laying performance at optimized inclusion levels [38]. Building on these findings, the present study systematically investigated the effects of acidifiers on antioxidant capacity, intestinal microbiota composition, and metabolic regulation in laying hens, with the aim of elucidating their roles in enhancing eggshell quality and yolk fatty acid composition. Collectively, these results provide a theoretical basis for targeted nutritional strategies to improve egg quality.

The composition and concentration of fatty acids in egg yolk critically influence egg physicochemical properties, flavor, and nutritional value, making them key indicators of egg quality [39]. Through LEfSe analysis, we identified 23 yolk fatty acids, of which 22 were significantly upregulated, whereas only DHA exhibited a decreasing trend, which has a nutritionally adverse change worthy of attention. The decline in DHA may primarily be attributed to the acidifier-induced reduction in intestinal pH, which may affect the micellisation process and transmembrane transport efficiency of long-chain polyunsaturated fatty acids in the intestinal lumen [40]. Secondly, short-chain fatty acids such as acetate and lactate present in the acidifier may outcompete long-chain PUFAs in intestinal absorption, thereby limiting DHA uptake [12]. In addition, alterations in the gut microbiota and the rebalancing of overall lipid metabolism may also place DHA at a competitive disadvantage in fatty acid deposition. Although the reduction in DHA represents an unfavourable change, the significant enrichment of the other 22 fatty acids suggests that acidifier supplementation exerts an overall positive effect on yolk lipid nutritional quality. The precise molecular mechanisms underlying the selective decline in DHA warrant further investigation. Notably, acidifier supplementation significantly increased the concentrations of seven fatty acids in egg yolks, including tetradecanoic acid, myristoleic acid, palmitic acid, palmitoleic acid, octadecanoic acid, cis-vaccenic acid, and arachidic acid. Among these, tetradecanoic acid and octadecanoic acid have been reported to possess antibacterial properties [41], suggesting that acidifier-induced fatty acid enrichment may contribute to improved egg microbial safety and shelf-life stability. Palmitic acid, a central substrate in sphingolipid biosynthesis, participates in sphingosine formation through the condensation of palmitoyl-CoA and serine catalyzed by serine palmitoyltransferase (SPT) [42,43]. Consistently, the elevated palmitic acid content observed in egg yolks was accompanied by the upregulation of sphingosine-related metabolites and serine metabolism pathways in our metabolomic analysis, indicating coordinated metabolic regulation. Moreover, the increased levels of myristoleic acid (C14:1) and palmitoleic acid (C16:1) reflect enhanced deposition of ω-7 monounsaturated fatty acids, which are recognized for their bioactive roles in improving lipid metabolism and cardiovascular health [44,45]. Supporting this, palmitoleic acid has been shown to alleviate hyperglycemia and hypertriglyceridemia while improving hepatic lipid metabolism in animal models [46]. The observed increase in cis-vaccenic acid was also consistent with previous reports, further corroborating the role of acidifiers in promoting the synthesis and deposition of health-beneficial fatty acids in egg yolks [30]. Collectively, these findings suggest that acidifier supplementation modulates yolk fatty acid composition through coordinated effects on intestinal environment, lipid metabolic pathways, and host-microbiota interactions, thereby enhancing the nutritional functionality of eggs.

In addition to improving yolk nutritional quality, acidifier supplementation markedly enhanced eggshell ultrastructure and mechanical integrity. Ultrastructural analyses confirmed that the eggshell is a highly organized biomineralized structure composed of the shell membranes, mammillary layer, palisade layer, vertical crystal layer, and cuticle [47]. Scanning electron microscopy revealed that eggshells from the acidifier-treated group exhibited denser calcite crystal packing and reduced mammillary spacing compared with those from the control group. Previous studies have demonstrated that decreased mammillary spacing is strongly associated with increased eggshell compressive strength and is considered a critical determinant of superior shell quality [48,49].The improvements in eggshell ultrastructure observed in this study were accompanied by elevated serum calcium and phosphorus concentrations, suggesting that the acidifier may enhance the intestinal availability of calcium and phosphorus, thereby providing an adequate mineral source for the dense deposition of calcite crystals in the eggshell. In the present acidifier formulation, formic acid constituted the highest proportion (36%), and its strong acidity effectively lowered intestinal pH, thereby promoting the dissolution and ionisation of calcium and phosphorus and enhancing their absorption efficiency [50,51]. Propionic acid may participate in active calcium transport by upregulating calcium-binding protein expression [51,52], while lactic acid and its salt, calcium lactate, can serve as a direct calcium source [53]. In addition, ammonium salts can release free acids under acidic conditions, further potentiating mineral solubility and absorption [54]. Beyond the reduction in mammillary spacing, acidifier treatment resulted in a denser and smoother eggshell cross-sectional morphology, characterised by a more compact and continuous crystalline network. These structural improvements coincided with elevated serum calcium and phosphorus concentrations, suggesting enhanced mineral availability. Collectively, we speculate that the acidifier may synergistically promote calcium and phosphorus mobilisation and utilisation through lowering intestinal pH, enhancing calcium-binding protein expression, and providing a directly available calcium source, thereby facilitating eggshell mineral deposition. This mechanism may provide the material basis for improved eggshell crystallisation, reinforce the physical barrier function, and ultimately reduce the risk of breakage.

Efficient eggshell formation depends on the intestinal absorption and metabolic utilization of calcium, phosphorus, and other essential minerals [55]. In this study, dietary acidifier supplementation markedly improved cecal morphology, as evidenced by well-organized villi, uniformly distributed goblet cells, and a significantly thickened lamina propria in the Exp group. These structural enhancements indicate improved intestinal integrity and absorptive capacity. Notably, serum metabolomics revealed an upregulation of taurodeoxycholic acid 3-glucuronide, a bile acid metabolite known to promote intestinal epithelial cell proliferation, enhance villus development, and protect the mucosal barrier against apoptosis [56]. Formic acid lowers intestinal pH and inhibits pathogen colonisation while promoting epithelial proliferation [26,57]. Propionic acid nourishes epithelial cells and suppresses pathogens [58]. Acetic acid serves as an energy substrate for colonic epithelial cells [59,60], and lactic acid promotes beneficial bacteria [61]. Ammonium salts release free acids under acidic conditions, exerting sustained antimicrobial effects [54]. These actions were associated with improved villus architecture and increased digestive enzyme activities, likely due to pH-activated enzyme function and stimulated pancreatic secretion [16,59,62]. Collectively, the acidifier formulation, dominated by formic acid with synergistic contributions from propionic, acetic, and lactic acids, enhances intestinal structure and digestive function, thereby facilitating nutrient absorption and indirectly supporting yolk nutrient biosynthesis and eggshell mineralisation.

Subsequent analysis of the cecal microbiota showed no significant differences in alpha diversity indices between the Con and Exp groups, indicating that dietary acidifier supplementation did not alter overall microbial richness or evenness. This suggests that the acidifier primarily modulated the relative abundance of specific taxa rather than reshaping global microbial diversity. Indeed, once a stable microbial ecosystem is established, dietary interventions may preferentially affect selected microbial populations without markedly changing overall diversity [63]. In contrast, beta diversity analysis revealed a clear separation between groups, demonstrating that acidifier supplementation significantly reshaped the cecal microbial community structure. Consistent with previous studies in poultry [64]. Bacteroidota and Firmicutes (including Firmicutes_A and Firmicutes_C) were the dominant phyla in both groups, together accounting for more than 60% of total sequences. Bacteroidota are known to facilitate the degradation of indigestible polysaccharides in the hindgut [65], whereas Firmicutes play a key role in host energy metabolism and digestive efficiency [66]. Notably, acidifier supplementation increased the relative abundance of Bacteroidota, indicating enhanced intestinal metabolic capacity [67]. In contrast, the reduced abundance of acetate-producing taxa such as Faecalibacterium may result from acidifier-induced alterations in intestinal pH and bile acid–mediated modulation of the luminal redox environment, to which these strict anaerobes are particularly sensitive.

At the molecular level, untargeted metabolomics revealed pronounced metabolic reprogramming induced by acidifier supplementation. VIP analysis showed that 2-S-Glutathionyl acetate and cholic acid were significantly upregulated in the Exp group, whereas 3-hydroxycinnamic acid was downregulated. The decrease in 3-hydroxycinnamic acid was consistent with the suppression of phenylalanine metabolism, indicating substantial alterations in amino acid metabolic pathways. KEGG pathway enrichment analysis corroborated these findings. In the Exp group, sphingolipid metabolism, arachidonic acid metabolism, tryptophan metabolism, and glycine, serine, and threonine metabolism were significantly enriched, reflecting an active metabolic state favoring lipid and protein biosynthesis [68,69]. These metabolic changes may be synergistically mediated by multiple components of the acidifier. Formic acid participates in one-carbon metabolism via the formate dehydrogenase pathway, providing one-carbon units for nucleotide and amino acid biosynthesis [25]. Propionic acid serves as a gluconeogenic precursor that can enter the TCA cycle to provide energy, while also regulating lipid metabolism through activation of the AMPK signalling pathway [23,24]. Acetic acid can be converted into acetyl-CoA, participating in the synthesis of fatty acids and cholesterol [70]. Lactic acid, as an important gluconeogenic precursor, can be converted into glucose in the liver or enter the TCA cycle for energy supply [71]. In contrast, pathways related to pyruvate metabolism, the tricarboxylic acid (TCA) cycle, oxidative phosphorylation, and phenylalanine metabolism were downregulated, indicating a shift in systemic energy utilization. Collectively, these metabolomic signatures identify potential biomarkers and metabolic pathways responsive to acidifier intervention.

The acidifier coordinately regulates lipid metabolism through propionic acid, which inhibits fatty acid synthase and activates AMPK, acetic acid, which provides acetyl-CoA, and formic acid, which participates in one-carbon metabolism [72], resulting in significantly increased serum HDL-C and decreased LDL-C. Although serum TC and TG were concurrently elevated, the increase in TC may partly derive from the HDL fraction itself, and the marked reduction in LDL-C, a well-established favourable change in atherosclerosis risk [73,74], may offset the potential adverse effects of modest TC and TG elevations, with the upregulation of 15-HETE further supporting a favourable modulation of lipid metabolism [75,76]. The synergistic enhancement of mineral utilisation and lipid metabolism jointly supports the physiological health of laying hens, as well as the improvement in yolk nutritional quality and eggshell structural integrity. The acidifier formulation, with a leading role of formic acid, synergistic contributions from propionic acid, acetic acid, and lactic acid, and sustained acidification by ammonium salts, underscores that the multi-component synergy serves as the key foundation for the observed integrated effects.

## 4. Materials and Methods

### 4.1. Ethics Approval Statement

All animal procedures were approved by the Animal Care and Use Committee of China Agricultural University (approval no. AW11215202-1-01, Beijing, China). The experiment was conducted in accordance with the Guidelines for the Use of Experimental Animals issued by the Ministry of Science and Technology of China.

### 4.2. Animals and Experimental Design

A total of 60 healthy 52-week-old Jingfen No. 6 laying hens with similar body weight and laying performance were selected for this study. After a 1-week adaptation period, the hens were randomly allocated to two treatment groups, with six replicates per group and five hens per replicate. Hens in the control group were fed a basal diet, the composition and nutrient levels of which are shown in Supplementary Table S1. Hens in the experimental group received the same basal diet and were supplemented with an acidifier through the drinking-water system. The acidifier was administered via the drinking water, and the dosage was adjusted daily to maintain the water pH at 6.0 ± 0.1 throughout the experimental period. The acidifier used in this study was formulated in our laboratory, consisting of a multi-component organic acid blend (formic acid 36%, propionic acid 10.8%, acetic acid 7.2%, lactic acid 6%, plus ammonium formate, ammonium propionate, calcium lactate, and water).

The experimental period lasted for 6 weeks. Throughout the trial, all hens were housed in an environmentally controlled facility maintained at 24–26 °C with appropriate ventilation. A lighting program of 16 h light and 8 h dark was applied. Feed and water were provided ad libitum throughout the experiment.

### 4.3. Sample Collection

On the final day of the experiment, blood samples were collected from the wing vein and centrifuged at 1500 rpm for 15 min at 4 °C. The serum was separated and stored at −80 °C for subsequent biochemical and antioxidant analyses. After a 12 h fast, hens were humanely euthanized by exsanguination and subjected to necropsy. Cecal tissue segments of approximately 0.5 cm were excised, gently rinsed with phosphate-buffered saline, fixed in 4% paraformaldehyde for 24 h, and then washed with running water for another 24 h before histological processing. Cecal contents were aseptically collected, immediately frozen in liquid nitrogen, and stored at −80 °C for 16S rRNA gene sequencing and digestive enzyme activity analysis. Eggs were randomly collected from each group for eggshell ultrastructural observation and yolk fatty acid composition analysis.

### 4.4. Laying Performance

Egg production and egg weight were recorded daily for each replicate throughout the experimental period. Feed consumption was recorded weekly by replicate. These data were used to calculate laying rate, average daily feed intake, egg mass, average egg weight, and feed-to-egg ratio. Egg mass was calculated by multiplying egg production by average egg weight. The feed-to-egg ratio was calculated as total feed intake divided by total egg weight for each replicate.

### 4.5. Serum Biochemical Indices, Antioxidant Parameters, and Digestive Enzyme Activity

Serum samples from all 60 hens were used for biochemical, antioxidant, and digestive enzyme assays. Serum concentrations of total protein (TP), albumin (ALB), alkaline phosphatase (ALP), aspartate aminotransferase (AST), high-density lipoprotein cholesterol (HDL-C), low-density lipoprotein cholesterol (LDL-C), triglycerides (TG), total cholesterol (TC), calcium (Ca), and phosphorus (P) were determined using commercial assay kits according to the manufacturer’s instructions (Nanjing Jiancheng Bioengineering Institute, Nanjing, China). Serum antioxidant parameters, including total superoxide dismutase (T-SOD), glutathione peroxidase (GSH-PX), catalase (CAT), and malondialdehyde (MDA), were measured using colorimetric assay kits according to the manufacturer’s protocols (Nanjing Jiancheng Bioengineering Institute, Nanjing, China). The activities of digestive enzymes in cecal contents, including lipase, chymotrypsin, amylase, and trypsin, were also determined using commercial assay kits following the manufacturer’s instructions.

### 4.6. Cecum Histopathology

Six hens per group were selected for histomorphological analysis. Cecal tissue samples were fixed in 4% paraformaldehyde, dehydrated, embedded in paraffin, and sectioned at a thickness of 5 μm. The sections were stained with hematoxylin and eosin (H&E) and Alcian blue according to standard protocols. Histological processing and staining were performed by Wuhan Servicebio Technology Co., Ltd. (Wuhan, China). Images were captured and analyzed using CaseViewer 2.4 software (3DHISTECH Ltd., Budapest, Hungary).

### 4.7. Eggshell Ultrastructural Analysis

Six hens per group were selected for eggshell ultrastructure analysis. Eggshell ultrastructure was examined using scanning electron microscopy (SEM). Briefly, two fragments of approximately 0.5 cm^2^ were carefully excised from the equatorial region of each eggshell. The eggshell fragments were mounted on specimen stubs using conductive adhesive and prepared for SEM observation. Cross-sectional images were obtained using a scanning electron microscope (SU8100, Hitachi Co., Ltd., Tokyo, Japan). For each fragment, three randomly selected fields were imaged under the indicated magnification conditions. The accelerating voltage was set at 3.0 kV, and images were acquired with field widths of 200 μm and 500 μm.

### 4.8. Determination of Egg Yolk Medium- and Long-Chain Fatty Acids

Six hens per group were selected for yolk medium- and long-chain fatty acids analysis. Egg yolk medium-chain fatty acids (MCFAs) and long-chain fatty acids (LCFAs) were determined using gas chromatography–mass spectrometry (GC–MS). Briefly, 500 μL of egg yolk sample was accurately measured and mixed with 1 mL of chloroform–methanol solution (1:1, *v*/*v*). The mixture was ultrasonicated at low temperature for 15 min, incubated at −20 °C for 15 min, and centrifuged at 13,000× *g* for 10 min at 4 °C. The lower organic phase was transferred to a 1.5 mL microcentrifuge tube and dried under a gentle stream of nitrogen. Subsequently, 0.5 mL of methanolic sodium hydroxide solution (0.5 mol/L) was added, vortexed for 30 s, and incubated at 60 °C for 30 min. After cooling to room temperature, 0.5 mL of n-hexane was added, vortexed for 30 s, and centrifuged at 13,000× *g* for 10 min at 4 °C. An aliquot of the upper hexane layer was transferred to a sample vial for GC–MS analysis. Peak areas were quantified using dedicated analytical software, and the concentrations of individual MCFAs and LCFAs were calculated by comparison with external standards.

### 4.9. 16S rRNA Gene Sequencing and Bioinformatics Analysis

Six hens per group were selected for 16S rRNA gene sequencing coupled with bioinformatics analysis. Total microbial genomic DNA was extracted from cecal content samples using the E.Z.N.A.^®^ Soil DNA Kit (Omega Bio-tek, Norcross, GA, USA) according to the manufacturer’s instructions. The quality and concentration of extracted DNA were assessed using 1.0% agarose gel electrophoresis and a NanoDrop 2000 spectrophotometer (Thermo Fisher Scientific, Waltham, MA, USA). DNA samples were stored at −80 °C until further analysis. The V3–V4 hypervariable region of the bacterial 16S rRNA gene was amplified using the primer pairs 341F (5′-CCTAYGGGRBGCASCAG-3′) and 806R (5′-GGACTACHVGGGTWTCTAAT-3′). PCR amplification was performed using a T100 Thermal Cycler (Bio-Rad, Hercules, CA, USA) under the following conditions: initial denaturation at 95 °C for 3 min; 27 cycles of denaturation at 95 °C for 30 s, annealing at 55 °C for 30 s, and extension at 72 °C for 45 s; followed by a final extension at 72 °C for 10 min. PCR products were verified by 2% agarose gel electrophoresis, purified using a PCR Clean-Up Kit (YuHua, Shanghai, China), and quantified using a Qubit 4.0 fluorometer (Thermo Fisher Scientific, Waltham, MA, USA). Purified amplicons were sequenced on an Illumina MiSeq platform (Illumina, San Diego, CA, USA) by Majorbio Bio-Pharm Technology Co., Ltd. (Shanghai, China).

### 4.10. Untargeted Metabolomics Data Processing and Analysis

Untargeted metabolomics analysis of serum samples was performed by Shanghai Majorbio Bio-Pharm Technology Co., Ltd. (Shanghai, China). Metabolite identification was conducted using an in-house database and public databases (HMDB, KEGG, and LIPID Maps), with matching parameters including retention time, mass accuracy < 10 ppm, and MS/MS fragmentation spectra. Differential metabolites were screened based on independent-samples t-test with false discovery rate (FDR) correction (q < 0.05), and metabolites with a fold change >1.2 or <0.83 were considered significantly different. Hierarchical clustering analysis was performed to generate heatmaps of differential metabolites, and Pearson correlation analysis was used to assess correlations among metabolites. Metabolic pathway enrichment analysis was conducted using the KEGG database with Fisher’s exact test, and *p* < 0.05 was set as the threshold for significant enrichment. All analyses and visualizations were performed using R packages (version 4.2.0).

### 4.11. Statistical Analysis

Data were analyzed using GraphPad Prism 9.5 software (GraphPad Software, San Diego, CA, USA). Each replicate was considered the experimental unit for performance-related parameters. Differences between the Con and Exp groups were analyzed using an unpaired Student’s t-test. For microbiota analyses, alpha diversity indices and the relative abundance of microbial taxa were compared between groups using appropriate statistical tests as implemented in the corresponding bioinformatics platforms. LEfSe analysis was performed to identify differentially abundant taxa, with an LDA score > 2.0 and *p* < 0.05 used as the threshold for significance. Data are presented as the mean with pooled standard error of the mean (SEM). Statistical significance was defined as *p* < 0.05, and highly significant differences were defined as *p* < 0.01.

Raw sequencing data were processed using the QIIME2 pipeline (version 2020.2). The DADA2 plugin was used for quality filtering, primer trimming, denoising, chimera removal, and paired-end read merging to generate amplicon sequence variants (ASVs). Representative ASV sequences were taxonomically annotated using the SILVA reference database (version 138). Alpha diversity indices, including Sobs, ACE, Chao1, and Shannon indices, were calculated using QIIME2. Beta diversity was evaluated by principal coordinate analysis (PCoA) based on Bray–Curtis dissimilarity. Differentially abundant taxa between the Con and Exp groups were identified using linear discriminant analysis effect size (LEfSe), with an LDA score > 2.0 and *p* < 0.05 considered statistically significant.

## 5. Conclusions

In conclusion, dietary acidifier supplementation was associated with improvements in egg quality in laying hens, as reflected by enhanced yolk fatty acid composition and reinforced eggshell ultrastructure. These changes were correlated with alterations in the intestinal microbiota, lipid and amino acid metabolism, and antioxidant capacity. Collectively, our findings indicate a strong association between acidifier supplementation and the observed improvements in egg quality, suggesting that acidifiers may represent a promising dietary strategy for producing nutritionally enriched eggs.

## Data Availability

The datasets generated and/or analyzed during the current study are available from the corresponding author upon reasonable request. The corresponding author will make the data available in a timely manner after receiving an appropriate request.

## Supplementary Materials

The following supporting information can be downloaded at: https://www.mdpi.com/article/10.3390/ijms27156859/s1.

## Abbreviations

The following abbreviations are used in this manuscript:

ACE	Abundance-based coverage estimator
ALB	Albumin
ALP	Alkaline phosphatase
AST	Aspartate aminotransferase
ASV	Amplicon sequence variant
Ca	Calcium
CAT	Catalase
CoA	Coenzyme A
Con	Control group
DADA2	Divisive Amplicon Denoising Algorithm 2
DHA	Docosahexaenoic acid
DNA	Deoxyribonucleic acid
Exp	Experimental group
GC–MS	Gas chromatography–mass spectrometry
GSH-PX	Glutathione peroxidase
H&E	Hematoxylin and eosin
HDL-C	High-density lipoprotein cholesterol
15-HETE	15-Hydroxyeicosatetraenoic acid
9-HPODE	9-Hydroperoxyoctadecadienoic acid
KEGG	Kyoto Encyclopedia of Genes and Genomes
LC–MS/MS	Liquid chromatography–tandem mass spectrometry
LCFAs	Long-chain fatty acids
LDA	Linear discriminant analysis
LDL-C	Low-density lipoprotein cholesterol
LEfSe	Linear discriminant analysis effect size
MCFAs	Medium-chain fatty acids
MDA	Malondialdehyde
MUFAs	Monounsaturated fatty acids
OPLS-DA	Orthogonal partial least squares discriminant analysis
P	Phosphorus
PCoA	Principal coordinate analysis
PCR	Polymerase chain reaction
PGE2	Prostaglandin E2
PGF2α	Prostaglandin F2 alpha
PUFAs	Polyunsaturated fatty acids
QIIME2	Quantitative Insights Into Microbial Ecology 2
SEM	Scanning electron microscopy; standard error of the mean
Sobs	Observed species
SPT	Serine palmitoyltransferase
TC	Total cholesterol
TCA	Tricarboxylic acid
TG	Triglycerides
TP	Total protein
T-SOD	Total superoxide dismutase
USD	United States dollar
VIP	Variable importance in projection
