# Supplementary material for: Dietary Acidifier Modulates the Gut Microbiota–Metabolic Axis to Improve Intestinal Health, Yolk Fatty Acid Profiles, and Egg Quality in Laying Hens
Source: Int J Mol Sci. 2026 Jul 30;27(15):6859. doi: 10.3390/ijms27156859 (PMC13467168; doi:10.3390/ijms27156859)
Supplement: Supplementary file 1 [file ijms-27-06859-s001.zip › ijms-4398394-supplementary.pdf]

Supplementary Table S1. Ingredient composition and nutrient content of basal diet (%, DM).

Ingredient	%	Nutrient and energyc[3]	
Corn	66.45	Chemical composition	
Soybean meal	22.80	AME, kcal/kg	2,700
Limestone	8.20	CP, %	15.52
Dicalcium phosphate	1.70	Lysine, %	0.75
DL-methionine	0.12	Methionine, %	0.37
Dicalcium phosphate	0.30	Methionine + cysteine, %	0.64
Choline chloride	0.10	Calcium, %	3.60
Mineral premix[1]	0.30	Total phosphorus, %	0.65
Vitamin premix[2]	0.03	Available phosphorus, %	0.39
Total	100.00		

1 Vitamin premix supplied (per kg of diet): vitamin A, 6,000 IU; vitamin D3, 1,500 IU; vitamin E, 15 IU; vitamin B1, 3mg; vitamin B2, 10.2 mg; folic acid, 0.9 mg; calcium pantothenate, 15 mg; niacin, 45 mg; vitamin B6, 5.4 mg; vitamin B12, 24μg; biotin, 150 μg;

2 Mineral premix provided (per kg of diet): Cu ($CuSO_4 \cdot 5H_2O$), 6.8 mg; Fe ($FeSO_4 \cdot 7H_2O$), 66 mg; Zn ($ZnSO_4 \cdot 7H_2O$), 83 mg; Mn ($MnSO_4 \cdot H_2O$), 80 mg; I (KI), 1 mg; Se (Na_2SeO_3), 0.3 mg;

3 Contents of VK, CP and Ca were analyzed. Contents of other nutrients and energy content were calculated based on Feeding Standard of Chickens.
